# Future thermal regimes for epaulette sharks (*Hemiscyllium ocellatum*): growth and metabolic performance cease to be optimal

**DOI:** 10.1038/s41598-020-79953-0

**Published:** 2021-01-12

**Authors:** Carolyn R. Wheeler, Jodie L. Rummer, Barbara Bailey, Jamie Lockwood, Shelby Vance, John W. Mandelman

**Affiliations:** 1 Anderson Cabot Center for Ocean Life, New England Aquarium, Boston, MA 02110 USA; 2 School for the Environment, The University of Massachusetts Boston, Boston, MA 02125 USA; 3 ARC Centre of Excellence for Coral Reef Studies, James Cook University, 1 James Cook Drive, Douglas, Townsville, QLD 4814 Australia; 4 Animal Care Division, New England Aquarium, Boston, MA 02110 USA

**Keywords:** Metabolism, Climate-change impacts, Animal physiology, Ichthyology

## Abstract

Climate change is affecting thermal regimes globally, and organisms relying on their environment to regulate biological processes face unknown consequences. In ectotherms, temperature affects development rates, body condition, and performance. Embryonic stages may be the most vulnerable life history stages, especially for oviparous species already living at the warm edge of their distribution, as embryos cannot relocate during this developmental window. We reared 27 epaulette shark (*Hemiscyllium ocellatum*) embryos under average summer conditions (27 °C) or temperatures predicted for the middle and end of the twenty-first century with climate change (i.e., 29 and 31 °C) and tracked growth, development, and metabolic costs both *in ovo* and upon hatch. Rearing sharks at 31 °C impacted embryonic growth, yolk consumption, and metabolic rates. Upon hatch, 31 °C-reared sharks weighed significantly less than their 27 °C-reared counterparts and exhibited reduced metabolic performance. Many important growth and development traits in this species may peak after 27 °C and start to become negatively impacted nearing 31 °C. We hypothesize that 31 °C approximates the *pejus* temperature (i.e., temperatures at which performance of a trait begin to decline) for this species, which is alarming, given that this temperature range is well within ocean warming scenarios predicted for this species’ distribution over the next century.

## Introduction

Ocean warming, resulting from global climate change, is already causing unprecedented changes to aquatic ecosystems worldwide^1^. The oceans absorb the Earth’s excess trapped heat that comes from anthropogenic emissions, and as such, sea surface temperatures are predicted to increase by as much as 4.0 °C under continued high emission scenarios^1^. These shifts are thought to have the most impact on tropical ecosystems where many species are adapted to narrow temperature ranges and also because seasonal temperature changes are small in comparison to those experienced in temperate zones^2,3^. Therefore, an organism’s ability to adapt to a change, whether by phenotypic or transgenerational plasticity, will become increasingly more important to surviving the changing conditions over the next century^2^. As most aquatic organisms are ectotherms, water temperatures regulate the biological and physiological processes that are crucial for survival^4^. Consequently, performance traits that reflect fitness (e.g., growth rates) exhibit thermal dependence, and this relationship can typically be depicted as a thermal performance curve^2,5^. In the context of ocean warming, establishing thermal performance curves for traits and species can help determine the suitability of future thermal habitats and help to predict both acute and chronic effects of warming on populations and ecosystems^6,7^.

The embryonic life stage in ectotherms represents a thermal bottleneck when it comes to climate change vulnerability^8,9^. Across ectotherms, embryos are generally less thermally tolerant than juveniles and non-reproducing adults. For example, embryos exhibit a narrower thermal performance curve—the difference between their maximum and minimum critical temperatures—and a lower thermal safety margin, which is the difference between their optimal temperature and their mean habitat temperature^9,10^. However, embryos, particularly of oviparous species, must also possess mechanisms to tolerate temperature changes throughout development because they cannot freely move and/or choose temperatures that are more favourable while *in ovo*^11^. Consequently, the mismatch between an increase in thermal vulnerability and the inability to behaviourally thermoregulate during the embryonic stages will likely have negative consequences for many aquatic ectotherm species in the face of ocean warming.

Chondrichthyan fishes (i.e., the class including sharks, rays, skates, and chimaera of which are mostly ectothermic) present a challenge in relation to ocean warming vulnerability, as many species are globally threatened (i.e., mainly from fisheries)^12^, poorly characterized in terms of basic life history^13^, have slow generation times and low reproductive output, and are logistically challenging for controlled laboratory experiments. However, these fishes have ecological, economical, and cultural significance worldwide, and require proper assessment and management, particularly with the rapidly changing marine environment^14^. Given that there are more than 1000 species of Chondrichthyans^12^, it is impractical to assume that comprehensive research investigating the effects of climate change can be conducted broadly. Therefore, we argue for a strategic focus on studies that investigate physiological tolerance limits and thresholds and to identify bioindicator species. Indeed, the indicator species concept is common in fields that include ecological and environmental monitoring to track the health of an ecosystem or to monitor the effectiveness of management^15,16^. However, it is less common to study an indicator species’ physiology—particularly via a thermal performance lens—for extrapolation within a taxonomic group, but that is the aim.

Because oviparous (egg-laying) chondrichthyans tend to be smaller in comparison to viviparous (live-bearing) species within this taxonomic group and are well suited to captivity, climate change impacts on *in ovo* growth and development have been studied in several species^17–20^. In fact, *in ovo* incubation time negatively correlates with increasing water temperatures in 28 species of sharks, skates, and chimaera that have been investigated to date^11^. Indeed, the maximum thermal limits for growth and development are imperative to understanding species and population level vulnerabilities. Therefore, given the similarities with regard to growth and development patterns, at least within oviparous species, an oviparous chondrichthyan may be ideal as an indicator species, especially for investigating physiological thermal tolerance with respect to climate change.

The epaulette shark (*Hemiscyllium ocellatum*), one such small oviparous shark endemic only to the Great Barrier Reef (GBR), Australia^21^, has been the focus of many climate change related laboratory studies^20,22–25^ because this species thrives in captivity and is considered of least concern in the wild by the International Union for Conservation of Nature (IUCN) Red List Criteria^26^. Additionally, *H. ocellatum* possess unique morphological and physiological traits that allow them to hunt in isolated tidal pools and survive extreme and repeated hypoxia conditions^27,28^. Therefore, this shark species may be resilient to challenging abiotic conditions, and as such, we propose that the *H. ocellatum* could serve as a conservative indicator species for chondrichthyans that are physiologically sensitive or logistically difficult to study. In other words, if epaulette sharks cannot cope with, in this case, thermal stress, how will other, less tolerant species fare?

Throughout the southern portion of their range on the GBR*, H. ocellatum* inhabit the reef flats where water temperatures seasonally vary, on average, from 21.7 to 27.9 °C^29^. While adult *H. ocellatum* do not use movement to behaviourally thermoregulate^30^, their maximum critical thermal limit (CT_max_)—the highest temperature at which acute survival is possible—is between 36 and 39 °C, which although seasonally-dependent, is well beyond temperatures that are predicted for their native range during the twenty-first century due to ocean warming^25^. Yet, this CT_max_ value only applies to adult survival and over short exposure periods (hours). Moreover, this value does not likely reflect the critical temperatures for other performance traits, such as growth and development, which would span longer periods (months to years). This value also does not necessarily apply to the more vulnerable life stages, such as embryos and neonates. Indeed, processes like growth and development that are most associated with early life stages are energetically more costly than acute survival alone; therefore, such growth and development traits may exhibit a narrower thermal range and more conservative thermal limits (multiple performances-multiple optima hypothesis^9,31^) than other traits and in other life stages. Given that rearing embryos and neonates at 32 °C has already resulted in significant mortality in this species^24^, we hypothesize that the *pejus* temperature range (temperatures at which performance traits begin to decline^32^) is just below 31 °C. To determine this, we investigated the effects of rearing temperatures including 27, 29, and 31 °C on growth, development, and physiological performance traits in epaulette sharks both *in ovo* and until 60 days post-hatch.

## Results

### Embryonic growth and development

**Figure 1:**
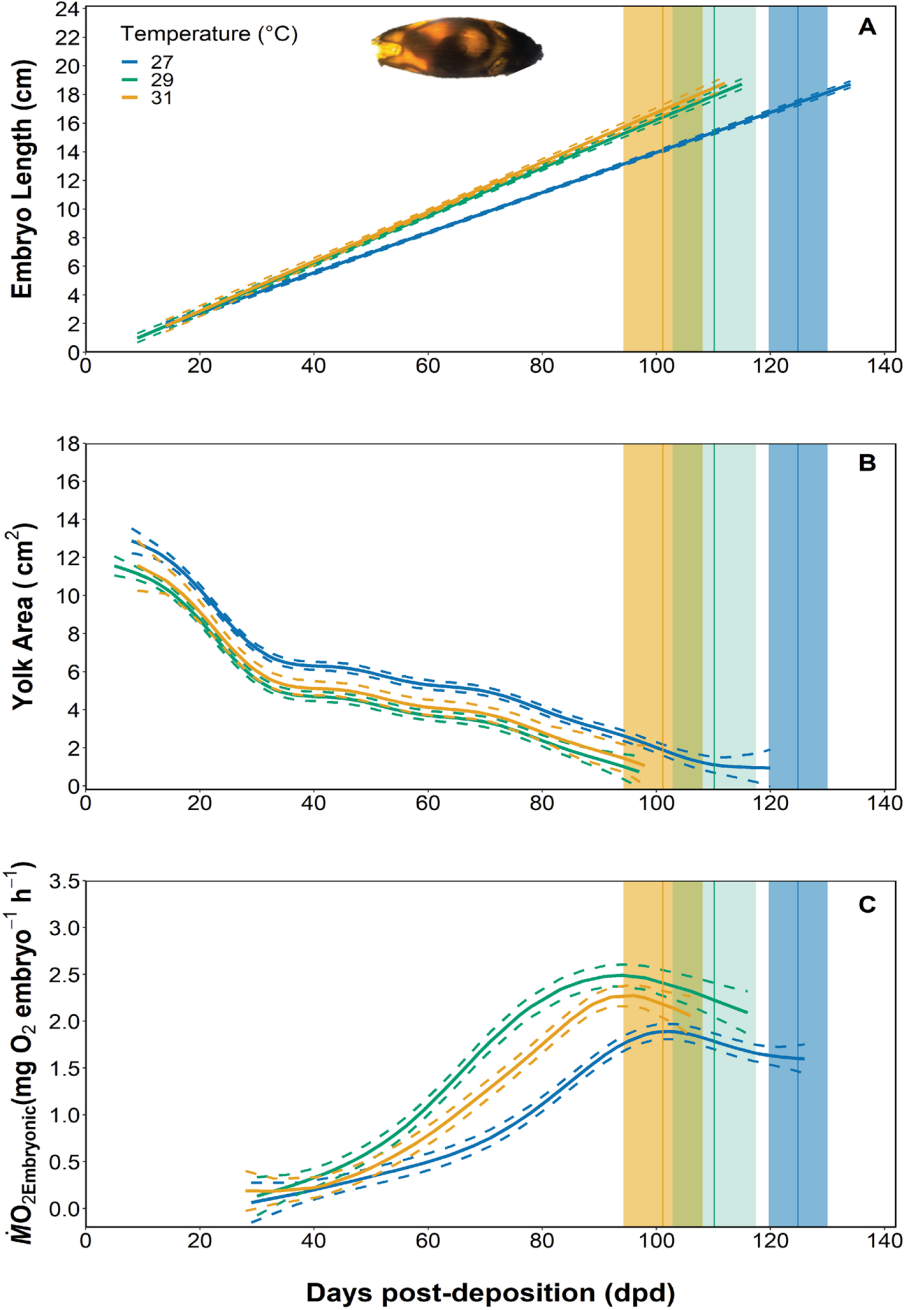
The growth rate (cm day^−1^) (**A**), yolk-sac consumption rate (cm^2^ day^−1^) (**B**), and embryonic oxygen uptake (mg O_2_ embryo h^−1)^ (**C**), over the *in ovo* incubation period at 27, 29, and 31 °C. The embryonic length data were fit using a mixed linear model (S1A), and yolk-sac consumption and RMR were fit with a generalized additive model (GAM) (S1B,C). Model fits are represented by solid lines, and 95% confidence intervals are represented by dashed lines. Model fits were considered significant at α = 0.05. The vertical lines represent the mean incubation time at each temperature treatment, and the shaded boxes represent the standard error around the mean.

Twenty-seven epaulette shark embryos were reared from 7 ± 3 days post-deposition (dpd) at either 27 (n = 14), 29 (n = 7), or 31 °C (n = 6) and monitored 2–3 times weekly to track embryo length and yolk area so that growth rates could be determined. Embryonic growth rates (cm day^−1^) were faster for embryos reared at both 29 and 31 °C when compared to embryos reared at 27 °C, but there were no differences in growth rates for embryos reared at 29 or 31 °C (Fig. 1A, adjusted R^2^ = 0.85, F_3,570_ = 1091, P = 0.022, see S1A). Correspondingly, yolk consumption (cm^2^ day^−1^) was also significantly faster in embryos reared at both 29 and 31 °C when compared to embryos reared at 27 °C (Fig. 1B, adjusted R^2^ = 0.721, F_3,609_ = 176.10, P < 0.0001, see S1B). Embryonic oxygen uptake rates (*Ṁ*O_2Embryonic_), which were used as a proxy for routine metabolic rates (RMR; mg O_2_ embryo^−1^ h^−1^), were measured bi-weekly throughout development to assess changes in metabolic costs in relation to temperature. RMR estimates were significantly different between embryos from all temperature treatment groups; specifically, RMR was higher in embryos reared at 29 °C when compared to those reared at 27 °C but then decreased in embryos reared at 31 °C (Fig. 1C, adjusted R^2^ = 0.756, F_3,193_ = 88.23, P < 0.0001, see S1C).

### Neonate survivorship and condition

**Figure 2:**
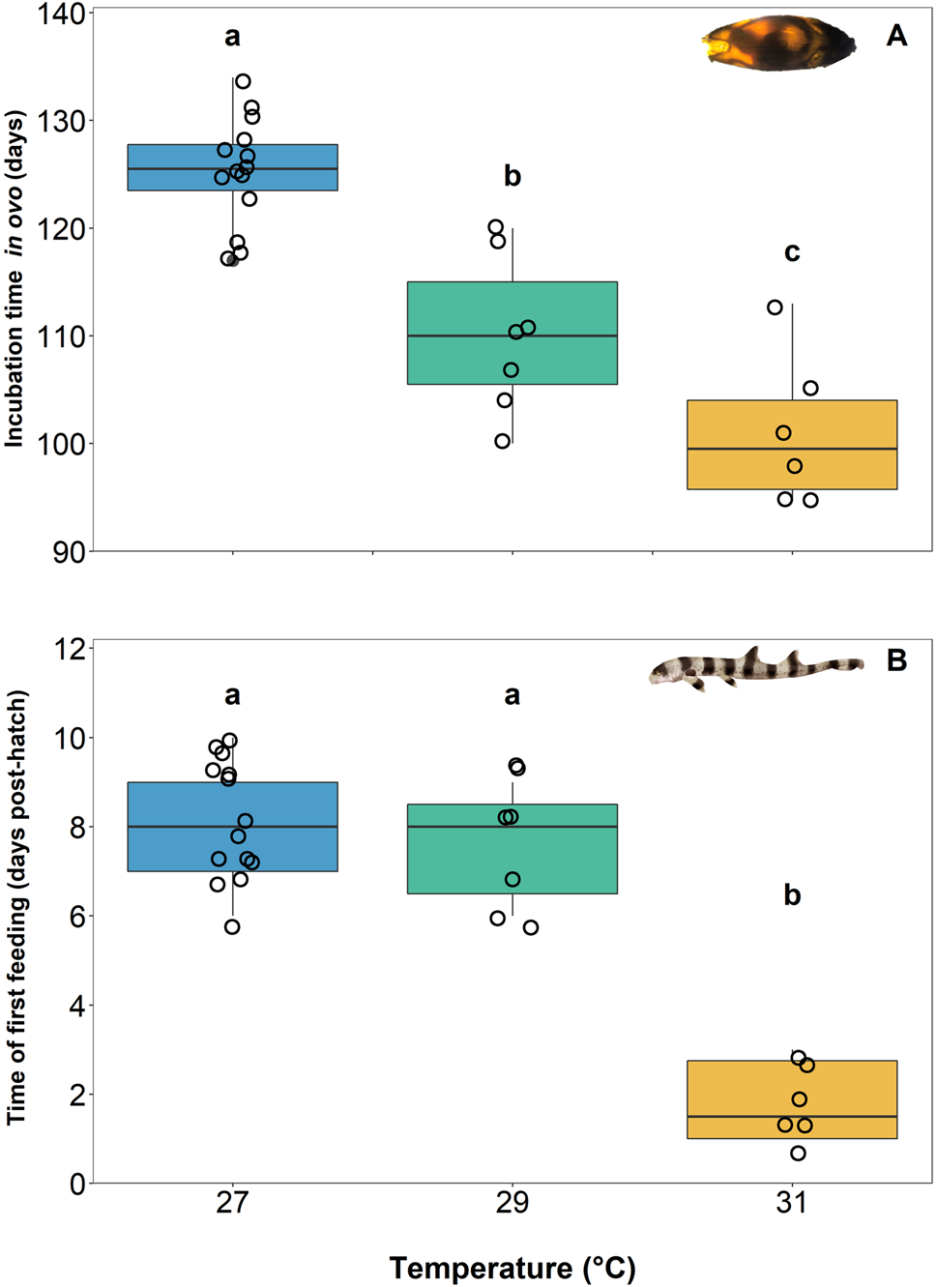
Mean incubation time to hatching (days) (**A**) and time of first exogenous feeding (post-hatch days) (**B**) across three treatment temperatures of 27, 29, and 31 °C. Boxes and whiskers represent the median, 25th, and 75th quartiles and 10 and 90th percentiles, respectively. The filled circles represent outliers, and open circles represent observations. Differing lowercase letters denote statistically significant differences at α = 0.05 (see S2).

**Figure 3:**
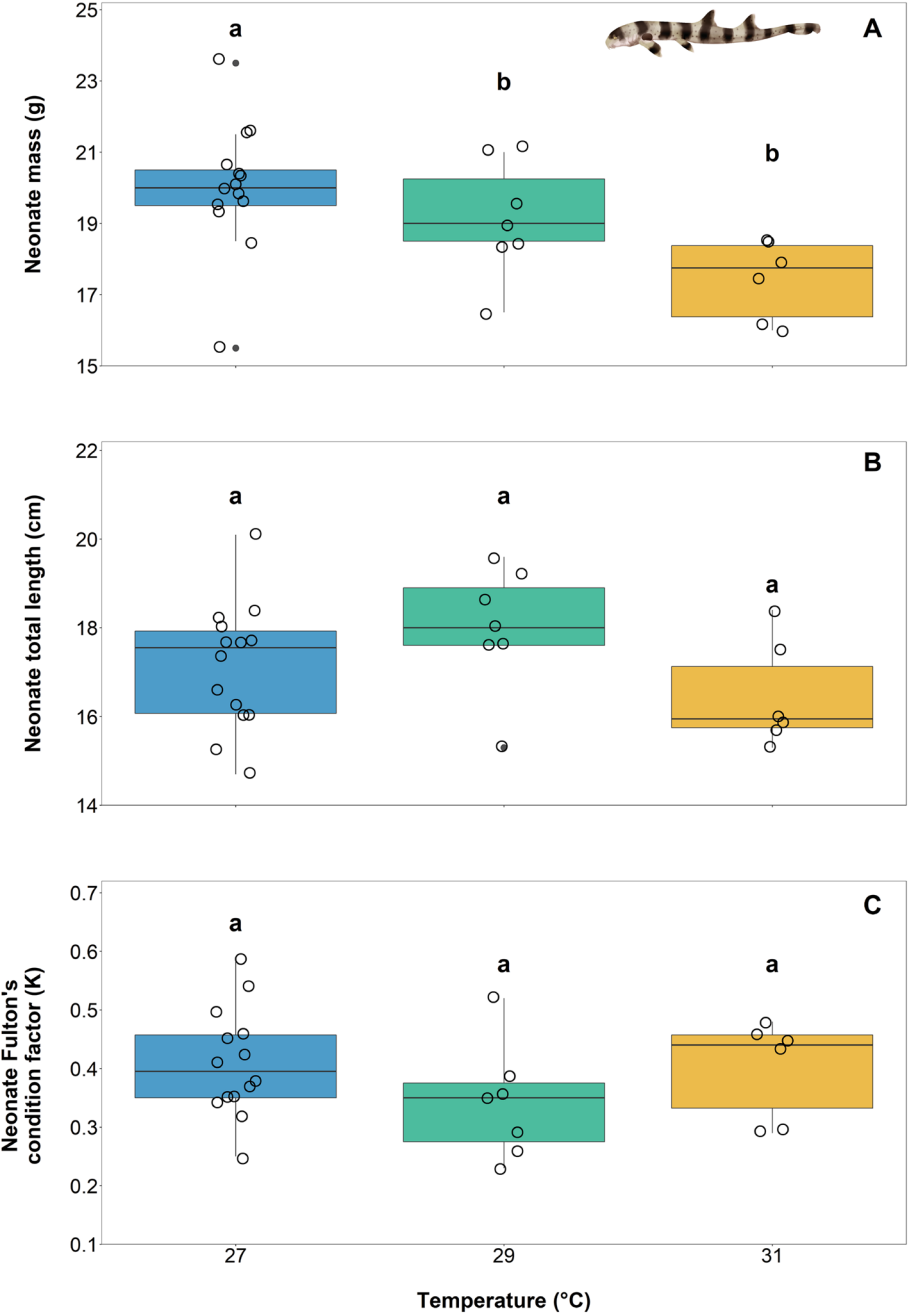
Mean (± SE) mass (g) (**A**), total length (cm) (**B**), and Fulton’s condition factor (K) (**C**) at hatching across three temperature treatments of 27, 29, and 31 °C. Boxes and whiskers represent the median, 25th, and 75th quartiles and 10 and 90th percentiles, respectively. The filled circles represent outliers, and open circles represent observations. Differing lowercase letters denote statistically significant differences at α = 0.05 (see S3).

Embryonic and neonatal survival was 100% throughout the study, irrespective of rearing temperatures. All except for two sharks—one reared at 27 °C and one reared at 29 °C—hatched during the dark photoperiod hours. Hatching time was significantly impacted by temperature as well; incubation time decreased from 125 ± 5 dpd to 110 ± 7 dpd and 101 ± 7 dpd in 27, 29, and 31 °C reared embryos, respectively (Fig. 2A, ANOVA; temperature, F_2,24_ = 38.04, P < 0.0001; see S2A).The fastest incubation (i.e., 95 dpd) occurred in 31 °C reared embryos. Neonates were significantly lighter in mass (i.e., by 2.6 ± 0.4 g) if the embryos had been reared at 31 °C, but no differences could be detected in neonate mass when embryos were reared at 27 or 29 °C (Fig. 3A, ANOVA; temperature, F_2,24_ = 5.561, P = 0.01037; see S3A). However, rearing temperatures did not affect neonate length or body condition (i.e., Fulton’s condition factor) (Fig. 3B, length ANOVA; temperature, F_2,24_ = 1.284, P = 0.2954, see S3B; Fig. 3C, Fulton’s condition factor ANOVA; temperature, F_2,24_ = 2.002, P = 0.1570, see S3C). Finally, the time it took for neonates to feed exogenously for the first time was 6 ± 1 days earlier for neonates reared at 31 °C when compared to neonates reared under the other temperature treatments (Fig. 2B, ANOVA; temperature; F_2,24_ = 55.28, P =  < 0.0001; see  S2B).

### Neonate metabolic rates

**Figure 4:**
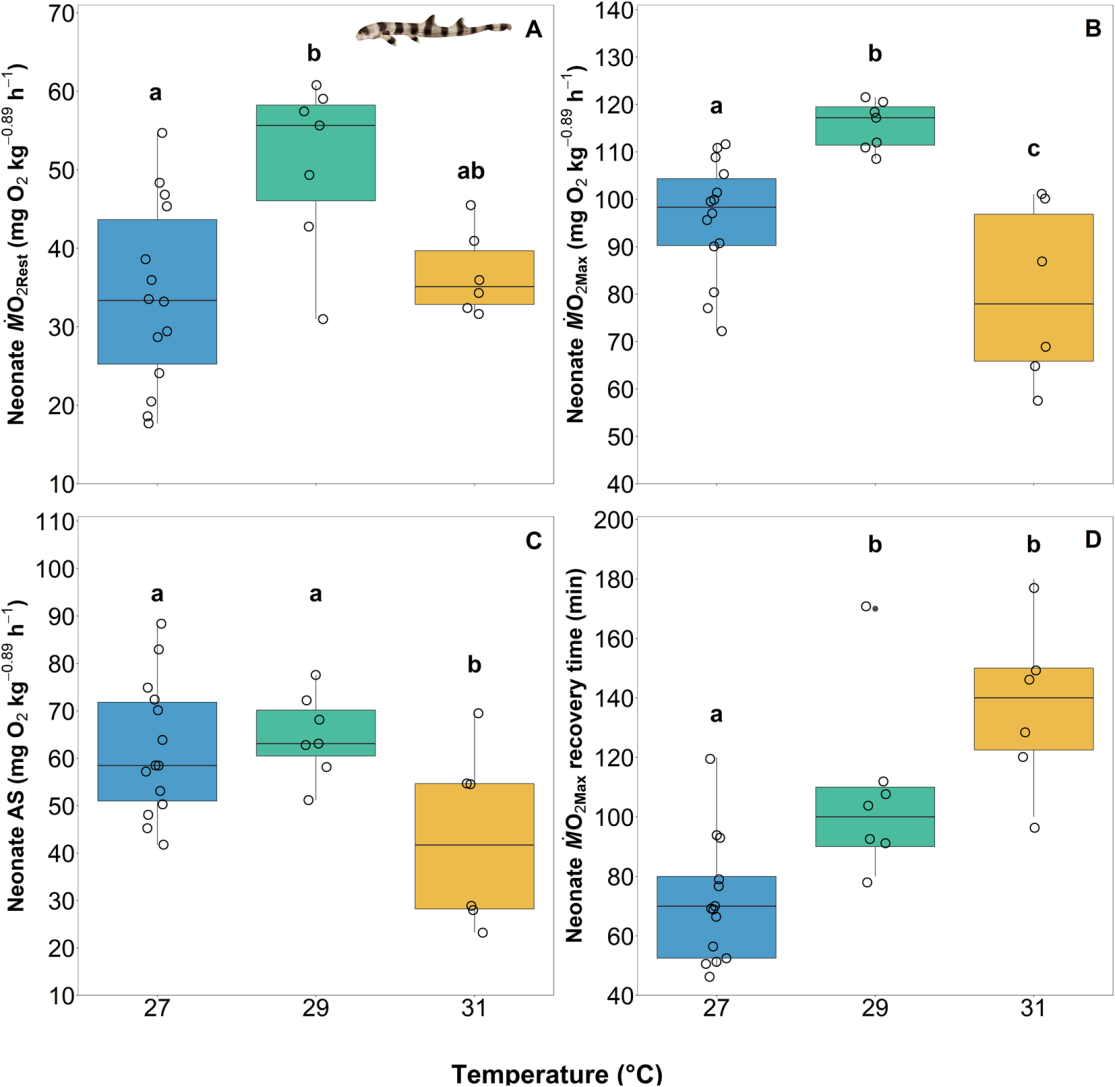
Boxplots of the *Ṁ*O_2Rest_ (mg O_2_ kg^−0.89^ h^−1^) (**A**), *Ṁ*O_2Max_ (mg O_2_ kg^−0.89^ h^−1^) (**B**), aerobic scope (AS) (*Ṁ*O_2Max_—*Ṁ*O_2Rest_; mg O_2_ kg^−0.89^ h^−1^) (**C**), and recovery time (min) from *Ṁ*O_2Max_ (**D**) for neonates across three rearing temperature treatments of 27, 29, and 31 °C. Boxes and whiskers represent the median, 25th, and 75th quartiles and 10 and 90th percentiles, respectively. The filled circles represent outliers, and open circles represent observations. Differing lowercase letters denote statistically significant differences at α = 0.05 (see S4).

Resting oxygen uptake rates (*Ṁ*O_2Rest_) and maximum oxygen uptake rates (*Ṁ*O_2Max_) were measured at 30- and 45-days post hatch, respectively, and as proxies for routine and maximum metabolic rates (RMR and MMR). In neonates reared at 29 °C, *Ṁ*O_2Rest_ was significantly higher than in neonates reared at 27 °C, but values for neonates reared at 31 °C were not significantly different than those from the other temperature treatments (Fig. 4A; ANOVA; temperature; F_2,24_ = 6.25; P = 0.006531; see S4A). A similar pattern was evident with *Ṁ*O_2Max_; however, in neonates reared at 31 °C, *Ṁ*O_2Max_ decreased below values observed in neonates reared under the other temperature treatments (Fig. 4B; ANOVA; temperature; F_2,24_ = 12.82; P = 0.0001635; see S4B). Aerobic scope (AS = *Ṁ*O_2Max_ − *Ṁ*O_2Rest_) was similar in neonates reared at under 27 and 29 °C treatments but decreased in neonates reared at 31 °C (Fig. 4C; ANOVA; temperature; F_2,24_ = 4.53; P = 0.02142; see S4C). Finally, there was a significant relationship between temperature and the time to recover following exercise (i.e., time from *Ṁ*O_2Max_ to *Ṁ*O_2Rest_); as rearing temperatures increased, recovery time increased (Fig. 4D, *Ṁ*O_2Max_ recovery ANOVA; temperature, F_2,19_ = 16.727, P < 0.0001; see S4D). Neonates reared at 27 °C required an average of 71 ± 5 min to recover from exercise; whereas, neonates reared at 29 °C and 31 °C required, on average, 107 ± 7 and 138 ± 6 min, respectively (Fig. 4D). There was nearly a doubling in recovery time with a 4 °C increase in rearing temperature.

## Discussion

**Figure 5:**
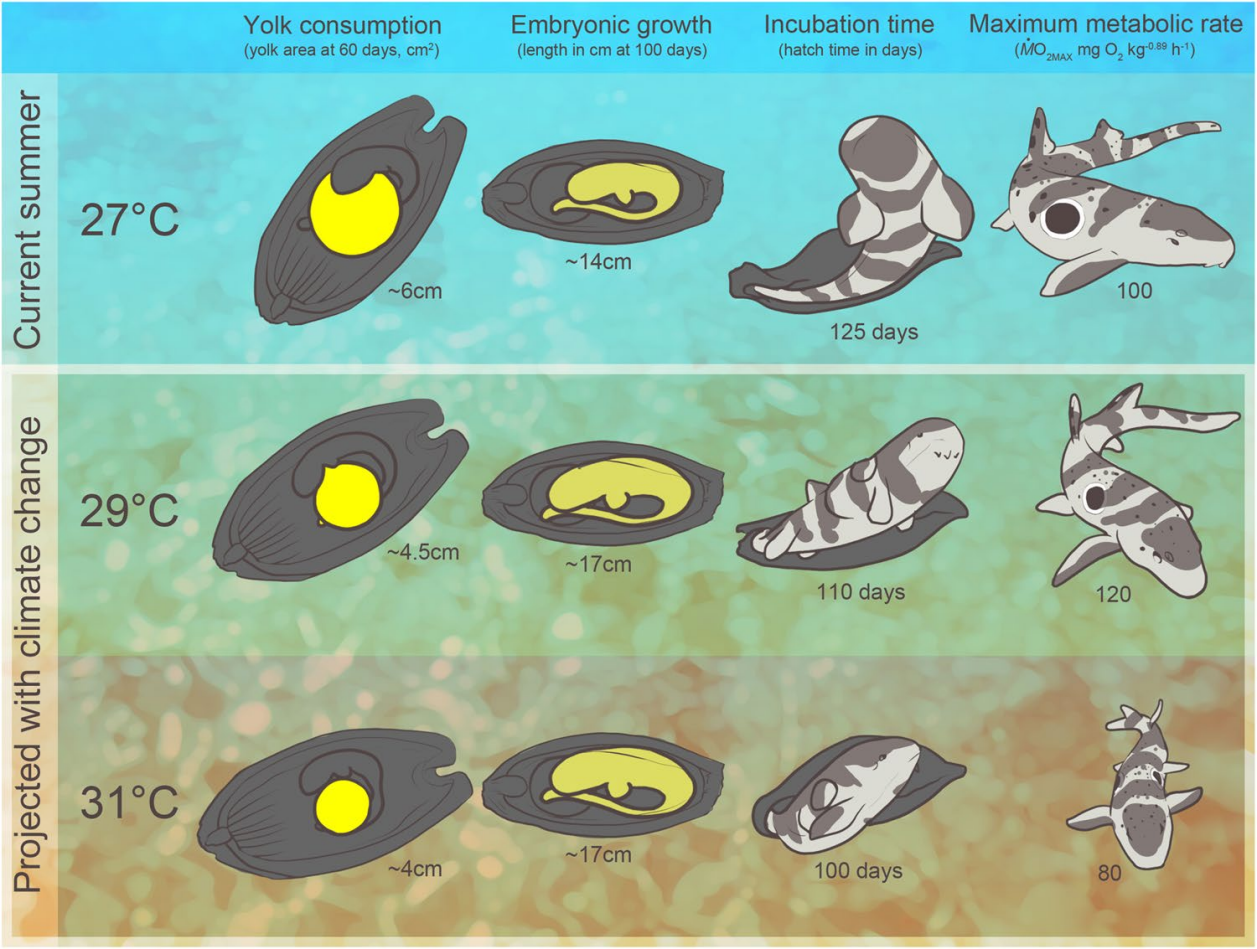
A conceptual diagram highlighting some of the main findings—including the yolk consumption and growth rate at mid-development, incubation time *in ovo*, and estimates of maximum metabolic rates at 45 days post-hatch—across current day (27 °C) and climate-change relevant (29 and 31 °C) temperature treatments. Illustration by E. Walsh.

Here, we investigated how growth, development, and metabolic performance traits of embryonic and neonate *H. ocellatum*, a tropical, shallow-water, oviparous shark species, were affected by increased temperatures relevant to mid and end-of-century ocean warming scenarios. We hypothesized that the *pejus* temperature for these traits was between 29 and 31 °C, which stems from previous research reporting high mortality of *H. ocellatum* offspring at 32 °C^24,25^. Our findings support this hypothesis. While survival was unaffected, embryos that were reared under elevated temperatures (i.e., 31 °C) grew more quickly, exhibited reductions in metabolic performance, and hatched at a lighter mass than their current temperature counterparts (Fig. 5). Furthermore, upon hatch, 31 °C-reared neonates exhibited a reduction in aerobic scope, indicating thermal impairment during this vulnerable life stage. Without phenotypic or transgenerational plasticity in these key traits, our findings could foreshadow biogeographic redistribution or changes in breeding season timing with future ocean warming scenarios, which could not only apply to epaulette shark populations on the GBR but also other similar species worldwide. This information is timely—especially given the rapid succession of mass coral bleaching events and other global heatwaves since the early twenty-first century—for inclusion in vulnerability assessments for the Great Barrier Reef as well as coral reef ecosystems worldwide.

While *H. ocellatum* has been investigated within the context of climate stress and environmental change since the start of the twenty-first century, it was only recently determined that prolonged exposure to elevated temperatures could be the most detrimental to this species, especially during early life stages. Indeed, previous research on *H. ocellatum* demonstrated that simulated ocean acidification conditions (elevated *p*CO_2_) have little impact on early growth, development, and survivorship of embryos and neonates^20^ and do not affect foraging behaviour, metabolic performance, or hypoxia sensitivity of adults^22,23^. Moreover, this species is noted as the most hypoxia and anoxia tolerant shark species studied to date^27,28^. In contrast, preliminary work to this study found that, when *H. ocellatum* were reared at 32 °C, survival to hatching was extremely low (37.5%), neonates exhibited irregular colouration and lacked their distinct black epaulette markings, and only one 32 °C-reared neonate from the study survived beyond three days post-hatch^24^. Furthermore, after the remaining neonate was transitioned back to 28 °C, proper patterns and colouration still did not develop^24^. Juvenile *H. ocellatum*—at least until 171 days post-hatch—were also negatively affected when acclimated to 32 versus 28 °C. Notably, survival and growth rates were reduced, despite maintained food consumption^25^. Based on these findings, we focussed only on the effects of elevated temperatures and chose 31 °C as the highest temperature treatment in an effort to observe only sub-lethal, if any, effects throughout our study.

Here*,* embryonic growth rates were tightly coupled with faster yolk consumption rates, presumably fuelling the accelerated growth rates that were also observed (Figs. 1A,B, 5). Interestingly, growth rates were similar in animals reared at 29 and 31 °C, perhaps indicating that the *pejus* temperature for this trait is between these temperatures, but nearing 31 °C. Johnson et al.^20^ reported slower growth rates during the early stages of embryonic development that increased after embryos had progressed to one third of their total incubation time. However, our data exhibited a linear relationship that did not reflect this temporal difference in growth rates.

The 31 °C-reared neonates in this study either hatched with more internal yolk reserves or consumed internal yolk stores more quickly during their final days *in ovo* when compared to their lower temperature reared counterparts. For oviparous chondrichthyan embryos, in general, the yolk transfers from the external yolk sac, through a yolk stalk, and then into an internal yolk sac that feeds into the spiral intestine where it is digested to provide energy for growth and development^33^. When the external yolk is depleted, there remains an internal reserve that feeds the late stage embryo for a short period of time (i.e., 5–7 days in epaulette sharks) and neonates for weeks to months after hatching^34^. The shift from endogenous energy sources (i.e., yolk) to exogenous feeding in neonates could therefore indirectly indicate the time at which the internal yolk has been consumed, thus allowing yolk consumption rates to be calculated. External yolk-sac consumption rates for 29 and 31 °C reared embryos were similar (Fig. 1B); the last visible signs of external yolk for embryos from both treatments was around 97 ± 2 dpd but closer to 118 ± 4 dpd for 27 °C-reared embryos. As such, hatching occurred significantly earlier (101 ± 7 dpd) in 31 °C-reared animals than in 29 °C-reared animals (110 ± 7 dpd) (Fig. 1B). From these findings, we hypothesize that there is a distinct relationship between internal yolk depletion and time of hatching, which is further supported by the time of first feeding. The 31 °C-reared neonates commenced exogenous feeding significantly sooner (Fig. 2B) than their lower temperature reared counterparts. One 31 °C-reared individual consumed food only 24 h after hatching. This overall finding regarding the fast rate of energy depletion *in ovo* and the early timing of exogenous feeding in 31 °C-reared epaulette sharks could have clear fitness implications relative to energy use, growth, and survival over the longer term.

Our findings regarding changes in embryonic *Ṁ*O_2_ (*Ṁ*O_2Embryonic_) represent, to our knowledge, the most fine-scale (6–12 data points per embryo) data set to date for a chondrichthyan species and especially over a climate change relevant temperature range. Estimates of RMR increased as temperature increased from 27 to 29 °C, demonstrating the regulatory role temperature plays on metabolic rate for ectotherms. However, *Ṁ*O_2Embryonic_ then declined in 31 °C-reared embryos when compared to those reared at either 29 or 27 °C. This could indicate a reduction in metabolic performance and/or a decrease in embryonic activity (tail-beating or ventilation) as an energy-saving strategy (Fig. 1C). Across treatments, *Ṁ*O_2Embryonic_ increased to a peak at 10–20 days prior to hatching and slightly decreased thereafter. The shape of this *Ṁ*O_2Embryonic_ curve has only been documented in one other oviparous chondrichthyan species^35^; all other studies report a continuous increase in RMR over development^17^. This decrease in O_2_ uptake rates during the final stages of development when the embryo has completely absorbed the external yolk sac (stage 34 from Ballard et al.^33^; stage 7 from Musa et al.^36^) could indicate crowding and mild hypoxia caused by the limited surface area for oxygen exchange across the egg case apertures and could underpin the mechanism signalling hatching.

The embryonic and neonate survival rates (i.e., 100%) throughout the present study imply that any negative effects of the rearing temperatures were only sub-lethal, at least during these early life stages. Incubation time was significantly impacted by temperature (Fig. 2A), which is a phenomenon that has been well-documented in development studies on ectothermic oviparous invertebrate and vertebrate species from both aquatic and terrestrial habitats^4,11,37^. Here, neonates had significantly lower mass if they were reared at 31 °C when compared to those reared at 27 °C, which supports the general hypothesis that warmer temperatures produce smaller offspring, a trend also observed across ectothermic invertebrate and vertebrate species alike^38^. Furthermore, the reduction in mass in 31 °C-reared animals further supports our hypothesis that expedited use of the internal yolk store may have, in part, signalled hatching. The reduction in body mass could be partially due to faster yolk consumption rates, which could compromise metabolic performance upon hatch.

At 30 days post-hatch, *Ṁ*O_2Rest_, which represents an estimate of SMR, increased in 29 °C-reared animals when compared to 27 °C-reared animals, and although not significant, decreased slightly in 31 °C-reared animals (Fig. 4A). A similar trend occurred for *Ṁ*O_2Max_, an estimate of MMR, which increased in 29 °C-reared animals when compared to 27 °C-reared animals, but was followed by a significant decrease in 31 °C-reared animals (Fig. 4B). As such, AS decreased in 31 °C-reared animals when compared to animals from the lower rearing temperatures. The increase in *Ṁ*O_2Rest_ between 27 and 29 °C-reared animals and decrease of *Ṁ*O_2Max_ between 29 and 31 °C animals indicates an overall reduction in metabolic performance, and that the *pejus* temperature likely falls between 29 and 31 °C (Figs. 4A,B, 5). The time required for 31 °C-reared animals to recover from the exercise protocol that was used to induce *Ṁ*O_2Max_ was twice the amount of time required for 27 °C-reared animals to recover (Fig. 4D). Collectively, these data indicate a decrease in the capacity for aerobic performance with elevate temperatures in neonates, which could translate to reduced survival beyond the 60 days post-hatch time period examined in this study.

Overall, we found that all metrics assessed, except length, Fulton’s condition factor, and *Ṁ*O_2Rest,_ were negatively impacted in 31 °C-reared animals. Embryos in this treatment group hatched more quickly (Fig. 2A), were lighter in mass (Fig. 3A), exhibited reductions in metabolic performance (decreased *Ṁ*O_2Max_ and AS; Fig. 4B,C), and required twice as long to recover from exhaustive exercise (Fig. 4D) than their lower temperature reared counterparts. Therefore, we conclude that thermal performance curves for the assessed traits for this species peak after 27 but before 31 °C, and would decrease thereafter, as supported by Gervais et al.^24^. While we recognize the genetic variation limitations that come with using only a single breeding pair, as was done in this study, we also note that the effects detected are, therefore, more likely solely due to temperature than genetic variability. To this end, more studies are needed, both within controlled laboratory conditions and amongst wild populations across latitudes.

Within an ecological context, epaulette sharks are distributed throughout the Great Barrier Reef in Australia and particularly occupy shallow reef flats where environmental conditions, such as dissolved oxygen and temperature, fluctuate quickly and often^29^. Heron Island (23.4423°S, 151.9148°E) in the southern Great Barrier Reef, has vast a reef flat where *H. ocellatum* are common. Given that peak egg deposition period at Heron Island is between August and November^29^, eggs deposited at the end of this time frame would develop on the benthos between November and February, when daily average temperatures peak at 28 °C. With mid- and end-of-century climate change predictions, it is likely that summer average temperatures could increase to upwards of 32 °C at this location. Gervais et al.^25^ found that juvenile *H. ocellatum* acclimated to 32 °C exhibited reduced growth rates despite maintaining constant food consumption rates. Moreover, these sharks behaviourally sought out warmer temperatures despite the fact that the thermal stress eventually resulted in 100% mortality. Overall, findings from Gervais et al.^24,25^ and the present study suggest that, under future ocean warming scenarios, *H. ocellatum* habitats could become too warm to support proper growth and development of embryos, neonates, and juveniles. The native range of *H. ocellatum* (i.e., the GBR) has already experienced heatwaves that have resulted in three widespread coral bleaching events (2016, 2017, and 2020^39,40^). These events, although acute, could sustain throughout the developmental window of this species, thereby greatly affecting reproductive success and therefore reducing the size of that particular year class of sharks. Indeed, both acute (e.g., a heatwave, a coral bleaching event) and chronic (i.e., ocean warming) thermal stress could be detrimental to individual year classes and population stability over the long term.

Overall, we concluded that, for *H. ocellatum*, a species otherwise resilient to severe changes in abiotic conditions, at least those investigated in isolation (e.g., acute exposure to temperature changes, ocean acidification scenarios, and extreme hypoxia and anoxia), the *pejus* temperature for development is between 29 and 31 °C. Because most oviparous sharks and skates are heavily dependent on the benthos, they are unlikely or unable to undertake large movements to more ideal temperatures^11,30^. Therefore, we expect these species to contract their geographical range to areas where thermal conditions remain optimal, but given the importance of mesopredators, this could come at the expense of ecosystem health. Indeed, species like the epaulette shark will be the ones to observe. Ultimately, our findings suggest that, under ocean warming scenarios for the middle and end of the twenty-first century, tropical oviparous chondrichthyan species will likely be exposed to their upper thermal limits for critical activities such as growth and development, which causes concern for the future health of the ecosystems they help to support.

## Materials and methods

### Ethics

All experimental protocols in this study were assessed and approved by the New England Aquarium Animal Care and Use Committee ethical code (protocol #: 2017-05), and furthermore conducted in accordance with all relevant guidelines and regulations.

### Embryonic growth and development

This work commenced in June 2017 and was completed in June 2019 at the New England Aquarium’s Animal Care Center (Quincy, MA, USA). Here, 27 epaulette shark egg cases were reared under three ecologically-relevant temperature treatments (27 °C: n = 14; 29 °C: n = 7, and 31 °C: n = 6) from 10 to 14 dpd until 60 days post-hatch. These eggs were collected from a singular breeding pair that had been maintained long term at the New England Aquarium (Boston, MA, USA). Then, all eggs were incubated in one of two 320-L aquarium systems, each fitted with a 250 L reservoir, UV sterilization, protein skimmer, two titanium heaters, and a 9:15 (d:n) hour photoperiod. Each egg case was suspended mid-water column with the proper polarity to ensure maximal success of development. All experiments were run either between June 2017 and May 2018, with a control 27 °C and experimental 29 °C temperature treatment, or between June 2018 and June 2019, where a replicated 27 °C and an experimental treatment of 31 °C were used. Temperature, salinity, ammonia, and pH were monitored daily, and nitrates and nitrites were measured weekly (Table 1).

**Table 1:** The mean (± standard error) water quality parameters measured daily (*) and weekly (**) throughout the study.

	Temperature (°C)*	pH*	NH_3_ (ppm)*	Salinity (ppt)*	Nitrites (mg/L)**	Nitrates (mg/L)**
System 1, Round 1	27.0 ± 0.03	8.18 ± 0.0	< 0.10	32.9 ± 0.16	0.06 ± 0.01	5.1 ± 0.2
System 2, Round 1	28.8 ± 0.04	8.19 ± 0.01	< 0.10	32.6 ± 0.16	0.14 ± 0.03	5.5 ± 0.25
System 1, Round 2	31.0 ± 0.03	8.23 ± 0.02	< 0.10	33.2 ± 0.22	0.04 ± 0.0	4.4 ± 0.1
System 2, Round 2	27.2 ± 0.07	8.19 ± 0.0	< 0.10	32.3 ± 0.14	0.04 ± 0.0	3.4 ± 0.23

The outer most fibrous layer of the egg capsule was carefully removed using a razor blade to allow for better viewing within the capsule without any negative effects on the embryos^20,41^. Eggs were candled 2–3 times per week in a 10-L aquarium after allowing five minutes of habituation following transfer. This was important, as embryos of oviparous sharks are known to cease movement when there is a perceived external threat^42^. Images and videos of each embryo were captured using a GoPro on narrow viewing setting so that growth rates (cm day^−1^) and yolk-sac consumption rates (cm^2^ day^−1^) could be tracked over time. Content was corrected for fish-eye bending around the edges, and ImageJ^43^ was used to measure embryo length as well as yolk length, width, and area to the nearest 0.1 cm.

### Embryonic oxygen uptake measurements

To quantify the metabolic cost of development in relation to temperature, oxygen uptake rates (*Ṁ*O_2_) of each embryo were measured *in ovo* using intermittent-flow respirometry. This was done every 2 weeks following the first 30 days of development because until ~ 30 days post deposition, the *Ṁ*O_2_ of the embryos was too small to detect while maintaining a chamber size large enough to accommodate the egg case. The whole respirometry setup comprised four glass chambers that were each 6 cm in diameter and 12 cm long (~ 339 ml volume) with baffled ends to allow even water flow. All four chambers were submerged in an 80-L water bath that received water directly from the animals’ respective aquaria to ensure identical water quality between holding conditions and test conditions. This water bath was also fitted with a standpipe so that the volume of water bath completely replenished 10 times per hour. Each chamber consisted of a flush pump (293.5 L h^−1^), which pumped water from the water bath, and a recirculating pump (302 L h^−1^) that circulated water throughout the chamber and through a glass segment consisting of an OXSP5 oxygen sensor spot connected to a fiber optic cable and a Firesting O_2_ system (Pyroscience, Aachen, Germany).

Embryos were carefully introduced into each respirometry chamber so that the egg case was never exposed to air. Upon transfer, chambers were covered with a small viewing window and were constantly supplied with filtered, oxygenated seawater for 2 h via the flush pump; this time period allowed embryos to habituate to the chambers following the transfer and found to be a sufficient period of time following preliminary trials^31^. After this 2-h habituation period, a relay timer was used to intermittently turn off the flush pump for 5, 10, or 15 min, depending on the stage of development; shorter measurement periods were used as the embryo developed. These time intervals were long enough to ensure that the decline in O_2_ could be detected but short enough such that O_2_ levels within chambers did not decrease below 80% saturation^44^. Following each this O_2_ uptake measurement period, the flush pump was turned on once again, thus returning O_2_ levels in the chamber water back to 100% air saturation; flush duration was always five minutes, regardless of embryo development stage. These measurement and flush cycles were repeated for 4 h (12–24 cycles depending on the measurement period) to ensure sufficient data points for each individual. After each respirometry trial, each embryo was returned to their respective holding conditions.

Respirometry chambers were also cycled empty for 30 min before and after each animal trial to account for microbial accumulation within each chamber; although, calculations demonstrated microbial respiration to be negligible (less than 2% of embryonic respiration). Respirometry chambers were also cycled with empty egg cases that had been maintained under the same conditions as the respective hatched neonates to account for the microbial respiration of each egg case; this too was also negligible (less than 3% of embryonic respiration). Oxygen uptake rates (*Ṁ*O_2_ in mg O_2_ embryo^−1^ h^−1^) were calculated as *Ṁ*O_2_ = SV where S represents the rate of change of O_2_ within the chamber, and V is the volume of the chamber^44^. Metabolic rates typically include a correction factor to account for body mass; however, because the embryos were maintained within the egg cases to determine incubation time to hatching, data were calculated as per whole embryo rates, similar to the protocols of Rosa et al.^17^. We did not correct for changes in mass using growth or yolk consumption rates, given that the former exhibited a linear and the latter a non-linear relationship (Fig. 1A,B); therefore, we could not interpolate changes in embryonic mass over development to use in *Ṁ*O_2_ calculations.

### Neonate survivorship, condition, and oxygen uptake measurements

Within 12 h of hatching, length (cm) and mass (g) of each neonate were measured, and the Fulton’s condition factor (K = mass/length^3^^45^) was calculated. Neonates were offered a mix of minced shrimp and clam, daily, after hatching to determine the time of first feeding (i.e., dph). Neonates were also provided pieces of PVC pipes for shelter. Neonate resting oxygen uptake rates (*Ṁ*O_2Rest_) were measured at 30 days post-hatch, as this allowed enough time post-hatch to ensure that feeding had switched from residual internal yolk stores to exogenously offered food. Prior to respirometry trials, however, neonates were fasted for 48 h to ensure they were in a post-absorptive state^22^. Neonates were weighed and introduced directly to their respective respirometry chambers, using the same intermittent-flow respirometry setup described for embryos, and then neonates where allowed to habituate to the chambers for 2 h. Then, the *Ṁ*O_2Rest_ for each individual was measured using a cycle consisting of 5 min of measurement and 5 min of flushing the chambers with clean, well-aerated seawater for a period of 4 h. Trials were always executed during daytime hours. Similar to embryos, this period was determined to be the shortest trial duration to ensure the individual was in a resting non-stressed state^31^.

Finally, maximum oxygen uptake rates (*Ṁ*O_2Max_) were measured in neonates at 45 days post-hatch using a standardized 3-min chase protocol with a 1-min air exposure^46^. This specific protocol and time were chosen because epaulette sharks are not continuous but rather burst swimmers; after 3 min of being chased, the neonate’s response changed from avoidance to a rolling behaviour. Neonates were then immediately placed in the same intermittent flow respirometry setup that was used for embryos. The initial slope of oxygen uptake was used to calculate *Ṁ*O_2Max_, and then neonates remained in the chamber until oxygen uptake rates returned to *Ṁ*O_2Rest_ levels measured for the individuals during their respective previous trial at 30 days post-hatch. A scaling exponent of 0.89 was used to correct the mass of each individual for the allometric relationship of mass and metabolic rate^47,48^. Finally, aerobic scope (AS) was calculated as *Ṁ*O_2Max_ − *Ṁ*O_2Rest_^31^.

### Statistical analysis

Mixed linear effects (embryo length) and generalized additive models (GAM; yolk consumption and RMR) using round (which encompasses the two rounds and systems) as a random fixed effect were applied to the data to determine if the rate of growth, yolk consumption, and oxygen uptake rates changed over time *in ovo* (see S1). All neonate data met all criteria of normality, non-multi-collinearity, and homoscedasticity from initial qq and residual plots and were analysed using a series of mixed linear models and one way analysis of variance (ANOVA) (see S2–S4). All statistical analyses were performed in RStudio (version 1.2.1335)^49^ where results were considered significant at α = 0.05.

## Supplementary Information

Supplementary Information.

## Data Availability

Data in this manuscript are available from the Research Data Repository (Tropical Data Hub) at JCU: 10.25903/YTXC-N035.
