# Supplementary material for: Future thermal regimes for epaulette sharks (Hemiscyllium ocellatum): growth and metabolic performance cease to be optimal
Source: Sci Rep. 2021 Jan 12;11:454. doi: 10.1038/s41598-020-79953-0 (PMC7804200; doi:10.1038/s41598-020-79953-0)
Supplement: Supplementary file 1 — Supplementary Information. [file 41598_2020_79953_MOESM1_ESM.docx]

**Supplemental Materials**

**­­­­­­Title:** Future thermal regimes for epaulette sharks (*Hemiscyllium ocellatum*): growth and metabolic performance cease to be optimal

**Authors:** Carolyn R. Wheeler^1,2,3^ (ORCID: 0000-0001-9976-8420);

Jodie L. Rummer^3^ (ORCID: 0000-0001-6067-5892);

Barbara Bailey^4^;

Jamie Lockwood^1^ (ORCID 0000-0002-5142-5071);

Shelby Vance^1^ (ORCID: 0000-0003-4140-5736);

John W. Mandelman^1,2^

^1^Anderson Cabot Center for Ocean Life, New England Aquarium, Boston, Massachusetts 02110, USA

^2^School for the Environment, The University of Massachusetts Boston, Boston, Massachusetts 02125, USA

^3^ARC Centre of Excellence for Coral Reef Studies, James Cook University, Townsville, Queensland 4814, Australia

^4^ Animal Care Division, New England Aquarium, Boston, Massachusetts 02110, USA

**Corresponding author:** Carolyn Wheeler: carolyn.wheeler23@gmail.com; 1 James Cook Drive

Douglas, QLD 4814 AUS

**S1. Figure 1.**

*A. Embryonic Growth Rates*

| **Mixed linear effects model** | | **Effects size:** 0.85 | | |
| --- | --- | --- | --- | --- |
| **Contrast (°C)** | **Estimate ± SE** | **df** | **t-ratio** | **p-value** |
| 27- 29 | -1.245 ± 0.182 | 570 | -6.856 | <0.0001 |
| 27- 31 | -1.582 ± 0.211 | 570 | -7.502 | <0.0001 |
| 29- 31 | -0.337 ± 0.238 | 570 | -1.416 | 0.3333 |

*B. Yolk-sac Consumption Rates*

| **Generalized additive model (GAM)** | | **Effects size:** 0.73 | | |
| --- | --- | --- | --- | --- |
| **Contrast (°C)** | **Estimate ± SE** | **df** | **t-ratio** | **p-value** |
| 27- 29 | 1.61 ± 0.157 | 598 | 10.245 | <0.0001 |
| 27- 31 | 1.181 ± 0.182 | 598 | 6.495 | <0.0001 |
| 29- 31 | -0.432 ± 0.205 | 598 | -2.108 | 0.0890 |

*C. Embryonic Metabolic Rates*

| **Generalized additive model (GAM)** | | **Effects size:** 0.77 | | |
| --- | --- | --- | --- | --- |
| **Contrast (°C)** | **Estimate ± SE** | **df** | **t-ratio** | **p-value** |
| 27- 29 | -0.601 ± 0.0735 | 184 | -8.19 | <0.0001 |
| 27- 31 | -0.363 ± 0.0751 | 184 | -4.83 | <0.0001 |
| 29- 31 | 0.600 ± 0.356 | 184 | 2.76 | 0.0176 |

**S2. Figure 2.**

1. *Incubation Time*

| **Contrast (°C)** | **Estimate ±SE** | **df** | **t-ratio** | **p-value** |
| --- | --- | --- | --- | --- |
| 27- 29 | 15.21 ± 2.80 | 24 | 5.431 | <0.0001 |
| 27- 31 | 24.19 ± 2.95 | 24 | 8.192 | <0.0001 |
| 29- 31 | 8.98 ± 3.37 | 24 | 2.666 | 0.0348 |

1. *Time of First Feeding*

| **Contrast (°C)** | **Estimate ± SE** | **df** | **t-ratio** | **p-value** |
| --- | --- | --- | --- | --- |
| 27- 29 | 0.571 ± 0.584 | 24 | 0.978 | 0.598 |
| 27- 31 | 6.31 ± 0.616 | 24 | 10.2 | <0.0001 |
| 29- 31 | 5.74 ± 0.702 | 24 | 8.17 | <0.0001 |

**S3. Figure 3.**

1. *Mass*

| **Contrast (°C)** | **Estimate ± SE** | **df** | **t-ratio** | **p-value** |
| --- | --- | --- | --- | --- |
| 27- 29 | 0.893 ± 0.746 | 24 | 1.197 | 0.4463 |
| 27- 31 | 2.619 ± 0.786 | 24 | 3.331 | 0.0076 |
| 29- 31 | 1.726 ± 0.896 | 24 | 1.926 | 0.1532 |

1. *Length*

| **Contrast (°C)** | **Estimate ± SE** | **df** | **t-ratio** | **p-value** |
| --- | --- | --- | --- | --- |
| 27- 29 | -0.836 ± 0.637 | 24 | -1.312 | 0.4022 |
| 27- 31 | 0.683 ± 0.671 | 24 | 1.018 | 0.5729 |
| 29- 31 | 1.519 ± 0.765 | 24 | 1.985 | 0.1377 |

1. *Body Condition (Fulton’s Index)*

| **Contrast (°C)** | **Estimate ± SE** | **df** | **t-ratio** | **p-value** |
| --- | --- | --- | --- | --- |
| 27- 29 | 0.0664 ± 0.0424 | 24 | 1.565 | 0.2797 |
| 27- 31 | 0.00762 ± 0.0447 | 24 | 0.170 | 0.9841 |
| 29- 31 | -0.05881 ± 0.0510 | 24 | -1.153 | 0.4919 |

**S4. Figure 4.**

1. *Ṁ*O_2Rest_

| **Contrast (°C)** | **Estimate ± SE** | **df** | **t-ratio** | **p-value** |
| --- | --- | --- | --- | --- |
| 27- 29 | -16.89 ± 4.84 | 24 | -3.488 | 0.0052 |
| 27- 31 | -2.82 ± 5.11 | 24 | -0.552 | 0.8464 |
| 29- 31 | -14.07 ± 5.82 | 24 | 2.418 | 0.0590 |

1. *Ṁ*O_2Max_

| **Contrast (°C)** | **Estimate ± SE** | **df** | **t-ratio** | **p-value** |
| --- | --- | --- | --- | --- |
| 27- 29 | -19.8 ± 5.92 | 24 | -3.350 | 0.0072 |
| 27- 31 | 15.9 ± 6.24 | 24 | 2.541 | 0.0455 |
| 29- 31 | 35.7 ± 7.11 | 24 | 5.017 | 0.0001 |

1. *Aerobic Scope*

| **Contrast (°C)** | **Estimate ± SE** | **df** | **t-ratio** | **p-value** |
| --- | --- | --- | --- | --- |
| 27- 29 | -2.93 ± 6.60 | 24 | -0.445 | 0.8972 |
| 27- 31 | 18.67 ± 6.96 | 24 | 2.684 | 0.0335 |
| 29- 31 | 21.60 ± 7.93 | 24 | 2.724 | 0.0307 |

1. *Ṁ*O_2max_ Recovery Time

| **Contrast (°C)** | **Estimate ± SE** | **df** | **t-ratio** | **p-value** |
| --- | --- | --- | --- | --- |
| 27- 29 | -35.7 ± 11.4 | 24 | -3.150 | 0.0116 |
| 27- 31 | -66.9 ± 12.0 | 24 | -5.598 | <0.0001 |
| 29- 31 | -31.2 ± 13.5 | 24 | -2.289 | 0.0768 |
